# Forging patterns and making waves from biology to geology: a commentary on Turing (1952) ‘The chemical basis of morphogenesis’

**DOI:** 10.1098/rstb.2014.0218

**Published:** 2015-04-19

**Authors:** Philip Ball

**Affiliations:** 18 Hillcourt Road, East Dulwich, London SE22 0PE, UK

**Keywords:** pattern formation, morphogenesis, developmental biology, chemical kinetics, reaction–diffusion, animal markings

## Abstract

Alan Turing was neither a biologist nor a chemist, and yet the paper he published in 1952, ‘The chemical basis of morphogenesis’, on the spontaneous formation of patterns in systems undergoing reaction and diffusion of their ingredients has had a substantial impact on both fields, as well as in other areas as disparate as geomorphology and criminology. Motivated by the question of how a spherical embryo becomes a decidedly non-spherical organism such as a human being, Turing devised a mathematical model that explained how random fluctuations can drive the emergence of pattern and structure from initial uniformity. The spontaneous appearance of pattern and form in a system far away from its equilibrium state occurs in many types of natural process, and in some artificial ones too. It is often driven by very general mechanisms, of which Turing's model supplies one of the most versatile. For that reason, these patterns show striking similarities in systems that seem superficially to share nothing in common, such as the stripes of sand ripples and of pigmentation on a zebra skin. New examples of ‘Turing patterns' in biology and beyond are still being discovered today. This commentary was written to celebrate the 350th anniversary of the journal *Philosophical Transactions of the Royal Society*.

## 1. Introduction

Let me begin my exploration of Alan Turing's paper [1] in what might seem an unlikely and unpromising place: the list of references. There are just six of these, and five are to books on rather broad and disparate topics: biological development, the permeability of membranes, the theory of elasticity and magnetism. Aside from standard textbooks, the only paper cited was canonical and almost 40 years old. It is true that many papers in the 1950s have a concise way with citations, but even so, this is a remarkably sparse list.

There are usually two reasons for such a state of affairs: either the authors are writing about a topic that no one cares (nor need care) about, or they have something of startling originality to say.

Turing's paper would hardly be in this collection if it did not fall into the latter category. But even by that measure, his contribution is extraordinary. While no one could reasonably assert that this paper has had as profound an impact on biology as that published a year later by Watson and Crick on the structure of DNA (which paper has?), it surpasses their achievement in this one regard: before Turing, no one had even really thought to ask the question that he poses.

At first it does not sound that way. ‘The purpose of this paper’, Turing writes, ‘is to discuss a possible mechanism by which the genes of a zygote may determine the anatomical structure of the resulting organism’ [1, p. 37]. Little new there, surely? The patterning and elaboration of an embryo, and how it is dictated by genes, are after all the matters discussed in the books by American zoologist Charles Manning Child and the British biologist Conrad Waddington that constitute two of the references. That body of work was, however, essentially experimental. Studies in the 1930s by Waddington and others had shown that some tissues can act as ‘organization centres' that, if implanted at a different location on the organism—or even sometimes on that of a different species—could induce the host tissue to undertake a new developmental path. Waddington spoke rather vaguely of a substance called an evocator that represents the ‘active principle’ of the organization centres; he seemed to regard it as a chemical compound of some kind. But how can chemicals, which simply diffuse through the fluid medium that contains them, give rise to order and structure?

That was the central question that Turing addressed. He presents a theoretical model in which chemicals that are diffusing and reacting may produce neither bland uniformity nor disorderly chaos but something in between: a pattern. This notion is, as we shall see, not entirely without precedent, but no one previously had thought to relate such an abstruse phenomenon to the question of biological growth and form—in short, to suggest how chemistry alone might initiate the process that leads from a ball of cells to a starfish, a horse or to us.

## 2. The codebreaker

A decade ago, the English mathematician Alan Turing, despite being an almost legendary figure to many scientists and the subject of an excellent biography [2], was largely unknown to the general public. If recent television dramatizations and stories about his wartime code-breaking work at Bletchley Park had not already changed that, then the 2014 biographical movie *The imitation game* has surely done so. The centenary of his birth in 1912 was marked with a number of commemorative events and eulogies, which highlighted in particular the shameful treatment he received after the war, precipitating what is generally thought to have been his suicide.

**Figure 1.**
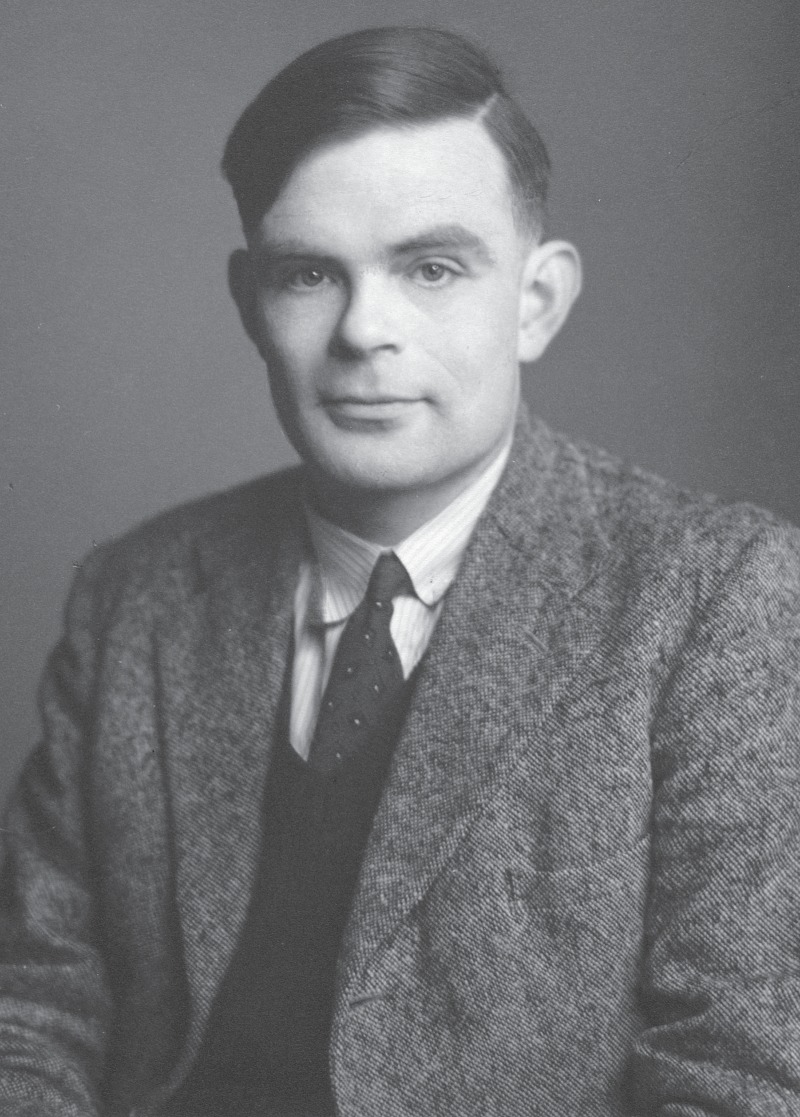
Alan Turing (1912–1954). Copyright © The Royal Society.

Turing (figure 1) studied mathematics at Cambridge, where he published perhaps his most important work at the age of just 24. In that paper, he showed that there exist some numbers that are not computable, meaning that they cannot be calculated as decimal numbers within a finite time. To make this argument, Turing needed to invoke the concept of an automatic ‘computing machine’, which is now regarded as a blueprint for the digital computer—a universal computing device that may store and execute programs.

When war broke out in 1939, Turing was enlisted at Bletchley Park, where he helped to crack the Enigma code used by the Germany Navy. His contribution was vital. ‘I won't say that what Turing did made us win the war’, one of his Bletchley colleagues said later, ‘but I daresay we might have lost it without him’ [3]. When the war ended, Turing moved to the National Physical Laboratory in London to assist with the construction of an electronic digital computer along the lines he had outlined. As it happened, the first such device was in fact built in Manchester in 1948, where Turing became the head of the Computing Machine Laboratory. During this period, he outlined the basics of what would later become known as artificial intelligence. Perhaps his best known contribution to this field is the so-called Turing test for determining whether machines can think. It involves a human interrogator who poses questions to the machine and to a human foil, and seeks to identify which is which. If there is no discernible difference in the responses, we have no logical reason to deny that the machine is thinking. This idea (adapted by the science fiction writer Philip K. Dick as the ‘Voigt-Kampff test’) was famously used in the opening sequence of Ridley Scott's 1982 movie *Blade runner* to identify non-human ‘replicants'.

Turing was actively homosexual at a time when this was illegal in Britain. In 1952 he was prosecuted, and his sexual orientation was deemed to pose a security risk in view of his wartime work (some of which remained classified for the rest of the century). He was sentenced to take a course of ‘corrective’ hormone therapy, and although Turing is said to have borne this sentence with ‘amused fortitude’, the shame and the physical effects of the hormone seem to have driven him to take his life in 1954 by biting on an apple laced with cyanide (some have asserted that his death was accidental). In 2009 the British prime minister Gordon Brown issued an apology for the way he was treated, saying that ‘on behalf of the British government, and all those who live freely thanks to Alan's work I am very proud to say: we're sorry, you deserved so much better’ [4]. During the 2012 centenary, a bill was introduced to the House of Lords asking for an official pardon for Turing's conviction for ‘gross indecency’, and in late December of 2013 the Queen signed a pardon, invoking the ‘royal prerogative of mercy’. There was much debate at the time about whether any kind of pardon can be appropriate for activities that are no longer, and never should have been, regarded as a crime. At any rate, at the time of his death, Turing was still highly productive, and the loss to British science was immense.

## 3. On growth and form

If you could have placed a bet, before the Second World War, on who might have asked the question that Turing posed, you would not have expected it to come from this mathematician. You should have been more likely to look to the Theoretical Biology Club of Cambridge University, a loose collection of innovative thinkers that included Waddington, J. Desmond Bernal, Joseph Needham and J. B. S. Haldane. All were sympathetic to the idea that biology might benefit from a reductive ‘physicist's' approach, rather than the purely statistical methods of classical genetics. They saw that the fundamental questions of biological development were about the generation of shape and form—of what was called morphogenesis—and Needham even approached the Rockefeller Foundation to fund a proposed Institute for Morphology. They recognized that the basic task was to understand how genetics, which they hoped might be ultimately understood at the atomic level, could give rise to aspects of symmetry and geometry in the whole organism. This notion of biological morphology was strongly felt in the first half of the twentieth century, not least because of the impact of the 1917 book by Scottish zoologist D'Arcy Wentworth Thompson, *On growth and form*, which is another of Turing's scant references. Thompson called for a more mathematical approach to biology that took account of the physical forces and constraints on an organism's development.

Turing left Cambridge in the year that the Theoretical Biology Club was formed, but he seems to have had no contact with Waddington's crowd while he was there, and it is not clear that he would have done so had he remained. All the same, the interests of this august group might have been expected to prepare the ground for a warm reception of a paper claiming to offer an explanation of the ‘chemical basis of morphogenesis’. That, on the contrary, Turing's paper was largely ignored for several decades is partly an ironic consequence of the fact that another Cambridge scientist with a physicist's pedigree—Francis Crick—reoriented interest in the ‘chemical basis' of developmental biology in another direction. For Crick and Watson's discovery of how genetic information may be encoded in DNA seemed at first to provide a more fruitful avenue for exploring the roots of growth and form. The task of decoding the molecular message of the genes, and the molecular mechanisms by which they interact and cooperate to orchestrate the cell, was to preoccupy biology for most of the second half of the century. It is only in recent years that this gene-centred view of biology has begun to reconnect with the mathematical and physico-chemical picture provided by Turing.

Quite aside from the biologist's notorious aversion to mathematics (there is plenty of it in Turing's paper, albeit not of a terribly advanced form, as he tried to reassure the reader), one can see why scientists familiar with the messy contingencies of cell cultures and assays might have been deterred from Turing's paper. Even he admits from the outset that ‘this model will be a simplification and an idealization, and consequently a falsification’ [1, p. 37]. Mentioning that at one point ‘the cells are idealized into geometrical points' [1, p. 37], Turing then begins to talk about Newton's laws of motion, elasticities and imaginary numbers in the kind of way that still today is apt to leave biologists despairing at the physical scientist's tendency to make biology abstract beyond all reach of reality.

Yet, the paper does not do that at all. Rather, it follows the physical scientist's instinct to express the basic problem in the simplest possible terms, and then to explore what the minimal requirements are of a model that captures the essential phenomena. Turing points out that the question of how an embryo develops and acquires shape and form—bilateral symmetry, say, and the budding of limbs—is one of *symmetry-breaking*. ‘An embryo in its blastula stage has spherical symmetry’, he writes [1, p. 41]. But how, if it consists simply of molecules randomly diffusing and reacting, can it ever escape that perfection? One might naively think that, as Turing writes, ‘It certainly cannot result in an organism such as a horse’—which he points out, in what looks like a sly meta-joke, ‘is not spherically symmetrical’ [1, p. 41]. (Whether or not this is an expression of Turing's playful nature, one of the paper's referees, J. B. S. Haldane, gently took him to task for occasionally belabouring the biologically obvious.)

Turing posits that among the molecular ingredients of this bundle of cells are components called morphogens (‘shape-formers'), which are somehow responsible for triggering development of a cell or tissue along a certain pathway. Waddington's vague chemical ‘evocators' of anatomical development were exactly what he had in mind here. Genes too, he said (and no one knew at that point quite what these inheritable entities were), are a ‘special class' of morphogen, albeit ones that are locked in the chromosomes and so are not free to diffuse through the cells. Hormones and skin pigments might also act as morphogens. All one really needs to know about them is that they diffuse and they react (triggering some developmental feature). This is why Turing's model is now known as an example of a reaction–diffusion system. It was not, however, the first such, as we shall see later.

Given the presence of these morphogens, one can see that there *are* deviations from spherical symmetry in the blastula. That is because random diffusion does not produce perfect uniformity: there are small, chance fluctuations in the concentrations of the substances, just as there are random local variations in national birth statistics or in the local temperature of a glass of water. Over time, these should average out and vanish—unless some process exists that will amplify such irregularities, so that they break the symmetry spontaneously and irrevocably.

Turing was not a physicist, and it is not clear that he realized that such symmetry-breaking by fluctuations was already well known in physical theory. It occurs, for example, in the behaviour of a magnet cooled through its so-called Curie point, where it switches from being non-magnetic to magnetic. Above the Curie point, the poles are randomly oriented; below it, they are aligned, creating a net magnetic field. But which way do they all point, given that any direction is equivalent to any other? The balance is tipped by chance fluctuations at the Curie point. In this way, symmetry (uniformity) is broken even though there is no driving force that specifies the direction in which it breaks. By the same token, a needle balanced on its tip (symmetrical along the vertical axis) breaks symmetry by falling over, even though no measurable force ‘pushes' it in any particular direction. Turing is, however, aware of this general kind of ‘unstable equilibrium’, to which he alludes with reference to a marble placed on top of a dome.

The question is then: into which kind of arrangements does a system of diffusing morphogens tip? What sort of patterns can we expect to see as a result? Much of Turing's paper is taken up with finding a mathematical answer to that question. To do so, he needed to assume a particular geometric arrangement of cells in which the morphogens move and interact. For the sake of simplicity, he chose to study a circular ring of cells. He wrote down equations describing the reaction and diffusion of morphogens, and showed that two situations might result. In both of them, the concentrations rise and fall around the ring in the form of waves. In one case, the waves are stationary, like acoustic standing waves: the peaks and troughs of concentration stay in the same place, creating a series of bands. In the other case, the waves are oscillatory, meaning that the peaks and troughs move around the ring: they are ‘travelling waves'.

**Figure 2.**
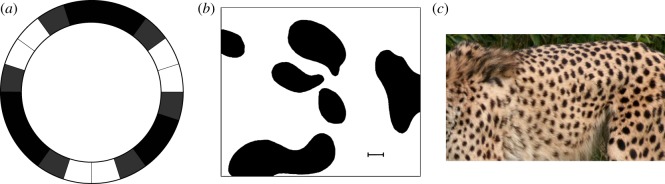
(*a*) The morphogen pattern in a ring of cells as deduced by Turing. The greyscale indicates concentration differences. (*b*) Turing's hand-calculated ‘dappled pattern’ created by a morphogen scheme in two dimensions [1, fig. 2]. (*c*) The resemblance to animal markings (here a cheetah) was obvious, albeit at this point no more than qualitative.

In fact, there may be several such waves of different wavelengths, and their interference can result in a series of ‘blobs' that differ from one another in size—a pattern, but not a perfectly regular one. Because he had to solve his equations by hand—not difficult, but laborious—Turing could offer only a rather sketchy indication of what the patterns would look like. One of them, in a 20-cell ring, is shown schematically in figure 2*a.* Turing showed this rather less strikingly as a histogram of concentrations; but a two-dimensional pattern that he calculated for a particular case of his reaction–diffusion system was much more evocative. It is shown in figure 2*b*, and one can see at once what it evokes: a dappled animal marking (see, for example, figure 2*c*), as Turing himself hinted.

Given his initial intention to explain embryonic morphogenesis, Turing focused the discussion of biological implications on the matter of how the scheme could account for the shapes of organisms that approximated his ideal geometries: for example, the sprouting of shoots as radial whorls in plants such as woodruff, or of tentacles on the cylindrical stems of the primitive aquatic animals called hydra. But, the glory of dappled things has excited more interest, and indeed Waddington wrote to Turing in 1952 saying that the best application of his theory would be ‘in the arising of spots, streaks and flecks of various kinds in apparently uniform areas such as the wings of butterflies, the shells of molluscs, the skins of tigers, leopards, etc’ [5]. Much of the focus of interest in chemical ‘Turing structures', as his stationary-wave patterns are now known, has subsequently turned to these pigmentation patterns as the ideal test-bed for whether Turing's theory stands up. I will turn to that shortly. First, it is apt to look more closely at why the theory, for all its originality, had precedents [6].

## 4. Chemical waves

The key attributes of the chemical system that Turing described, in which travelling or stationary waves in concentration can arise from a combination of reaction and diffusion, had already been discussed more than 40 years earlier. In 1910, the Austrian–American ecologist and mathematician Alfred Lotka described a chemical reaction that could undergo oscillations of this kind [7]. Lotka was not particularly interested in chemistry *per se*. For him, the reaction was an analogy for the dynamics of animal populations, and the collisions and reactions that may take place between molecules were just proxies for the interactions between predatory beasts and their prey. Chemical ingredients can be ‘consumed’ by reactions just as prey can be consumed by their predators. And both predators and prey can multiply their own kind by reproduction.

This last aspect is the crucial one: an ingredient of the process can produce more of itself. This is possible for a chemical reagent if it is autocatalytic, which means that it acts as a catalyst that speeds up the rate of its own formation. Autocatalysis is a positive feedback process: the more that is made, the faster it appears. That kind of blow-up allows small, random fluctuations in concentration to be amplified into large non-uniformities—into the ‘patches' that Turing's process can generate. Unchecked, positive feedback will blossom out of control, at least until the ingredients that feed it are exhausted. For chemical reactions, fresh reagents are brought into play at any location when they diffuse there, and so the outcome depends on a delicate balance between the rates of reaction and the rates of diffusion.

Lotka's paper described only a chemical system in which oscillations are transient—eventually they die out, like the damped oscillations of a ringing bell. But 10 years later he outlined conditions under which they could persist [8]. And in 1921, chemical oscillations were reported by William Bray of the University of California at Berkeley in a real reaction, that between hydrogen peroxide and an iodate salt to produce iodine. Although Bray was a distinguished chemist, no one knew what to make of his observations and they were largely ignored.

Travelling wavefronts resulting from a reaction–diffusion process were also identified in the 1930s by the geneticist Ronald Fisher, who devised a mathematical model [9]—extended by the Soviet mathematical physicist Andrei Kolmogorov [10] and now called the Fisher–Kolmogorov equation—describing how an advantageous allele spreads in a population. This model is now regarded as a very general prescription for a certain type of reaction–diffusion process, but at the time, with theoretical biology in its infancy (Fisher was one of the pioneers), there was little appreciation that an approach devised in one area of biology (population genetics) should have anything to say about another (morphogenesis).

In this respect, Lotka's perception that population dynamics could be rephrased as a problem of chemical kinetics was deeply insightful, and foreshadowed Turing's own recasting of a biological problem as a chemical one. It was also prescient, for at the same time that Turing was working out a theory that could account for pattern-forming reaction–diffusion processes, the first chemical system of this kind to receive serious attention was being discovered. In the 1950s, the Soviet biochemist Boris Belousov devised a cocktail of reagents as a simplified model of the process in which sugars are broken down in the body, and he found that the mixture oscillated back and forth between two states. His claims were met with scepticism, because they seemed at face value to violate the second law of thermodynamics, which can be interpreted as stipulating that any chemical process can only proceed in one direction. Specifically, the second law states that all change happens in the direction that increases the net entropy of the universe—this, you might say, is the ‘downhill’ direction. Belousov's results appeared to be tantamount to saying that his reaction was ‘downhill’ in both directions, which made no sense. Sadly for Belousov, no one had noted what Lotka had already made plain decades earlier, which was that thermodynamics can tell us only about the stable, unchanging equilibrium state of a system. If, Lotka said, a system is provided with an unceasing flux of energy and ingredients, it can be driven away indefinitely from an equilibrium state: it becomes a non-equilibrium system. Then, classical thermodynamics is silent about the outcome. The oscillating chemical reactions of both Bray and Belousov will, if left alone, eventually exhaust themselves and settle into an equilibrium state. But this can take a long time, and there is no thermodynamic prohibition to their changing direction on the way. Moreover, if these reactions are constantly provided with fresh ingredients, and if the reaction products are carried away, the oscillations continue indefinitely, persisting in what is then a permanently out-of-equilibrium system.

In the absence of this understanding, Belousov's results were dismissed and he was barely able to publish them. But in the 1960s they were explored by Anatoly Zhabotinsky, a graduate student at Moscow State University, who discovered a variation of Belousov's mixture that would switch back and forth between red and blue. Such dramatic contrasts were impossible to deny, and Zhabotinsky gradually won acceptance within the Soviet Union that oscillating chemical reactions are real. When he discussed these findings at an international conference in Prague in 1967, chemists in the West were intrigued and began to figure out what was going on. During the 1970s, the mechanism of the so-called Belousov–Zhabotinsky (BZ) reaction was decoded, and it came to be seen that autocatalysis of some of the intermediate molecular reagents was the key [11].

**Figure 3.**
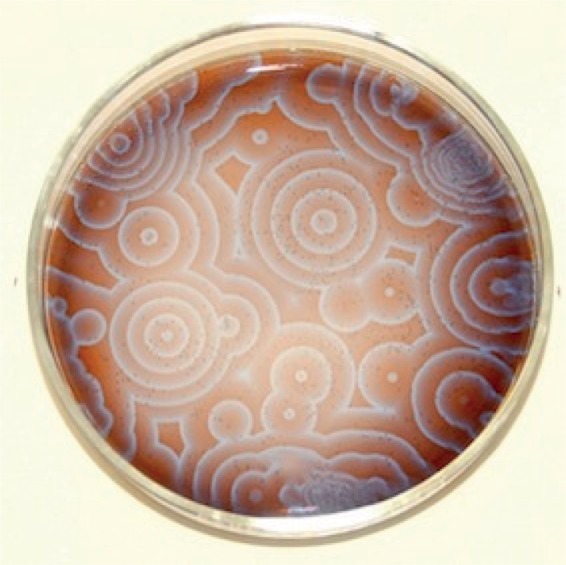
Patterns in the Belousov–Zhabotinsky reaction. Image courtesy of Michael C. Rogers and Stephen Morris, University of Toronto.

Moreover, it became recognized that the BZ reaction could produce patterns not just in time—the red–blue oscillations—but in space too. In a well-mixed solution, the colour changes all at once. But, if the reaction mixture is placed in a shallow dish, and particularly if the diffusion of molecules is slowed down by infusing the chemical mixture in a gel, the colour change may be initiated at a particular location, thanks to some random fluctuation in concentration or another disturbing influence, from which it radiates as a ‘reaction front’—a travelling wave of a different colour from its surroundings. And because the reaction oscillates, subsequent waves follow at regular intervals, producing a series of concentric rings like ripples in a pond. Where these wavefronts touch, they annihilate one another. Other triggers can elicit spiral waves instead, making the BZ reaction a rich source of chemical patterns (figure 3) [12].

All the same, the BZ reaction is *not* quite the kind of phenomenon sketched by Turing that generates travelling waves. They are all variants of pattern-forming reaction–diffusion processes, but the states they produce depend on the details: on the relative rates of reaction and diffusion, for example, and the nature of the reactions themselves. Given the highly mathematical and abstract nature of Turing's analysis, none of this was clear from his paper; indeed, it is almost devoid of any intuitive, physical picture of how the patterns arise, and one can only speculate about whether Turing had such a picture himself, or deemed it necessary. His reaction–diffusion equations are not wholly consistent with a real molecular picture, for instance—they can lead to negative concentrations, although Turing prohibited this by fiat.

The qualitative essentials of his model did not emerge until two decades later. In 1972, developmental biologists Hans Meinhardt and Alfred Gierer at the Max Planck Institute for Virus Research in Tübingen, Germany, devised a theory of biological pattern formation caused by diffusing reagents that paralleled Turing's [13]. They did not knowing about Turing's work until a referee of their paper pointed it out (Meinhardt 2012, personal communication). In Meinhardt and Gierer's model, stationary chemical patterns can result from two interacting ingredients—equivalent to Turing's morphogens—if they have specific characteristics. One is an ‘activator’, which is autocatalytic and so introduces positive feedback. The other is an ‘inhibitor’, which suppresses the autocatalysis of the activator. Crucially, they must have different rates of diffusion, the inhibitor being faster. In effect, this means that the activator's self-amplification is corralled into local patches, whereas the inhibitor prevents another such patch from growing too close by. Meinhardt and Gierer found that Turing's equations describe just this situation [14]. By a curious coincidence, a theoretical model similar to this activator–inhibitor scheme was also proposed in 1972 for a predator–prey system [15], although it tends to be unjustly overlooked today.

**Figure 4.**
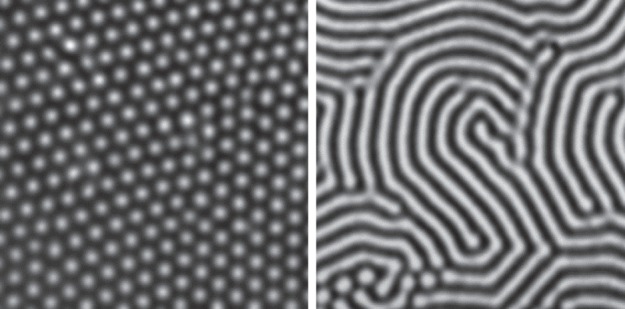
The generic patterns of an activator–inhibitor scheme. Images: courtesy of Jacques Boissonade and Patrick De Kepper, University of Bordeaux.

The availability of computers made it easier to deduce what are the generic patterns produced by activator–inhibitor systems: they generate quasi-ordered spots and stripes, with pattern features all of roughly the same size and separation (figure 4). These outcomes—a chemical leopard and chemical zebra—made it all the more plausible that Turing patterns might explain animal markings. In the 1980s, Meinhardt and mathematical biologist James Murray at the University of Washington in Seattle worked independently to show that Turing's theory offered a possible explanation for a wide range of animal pigment patterns, from zebras to giraffes to seashells [16–18]. The idea here is that the morphogens turn on or off genetic pathways that stimulate the production of pigments—in mammal skins this is the pigment melanin, which generates colours from tawny to black.

**Figure 5.**
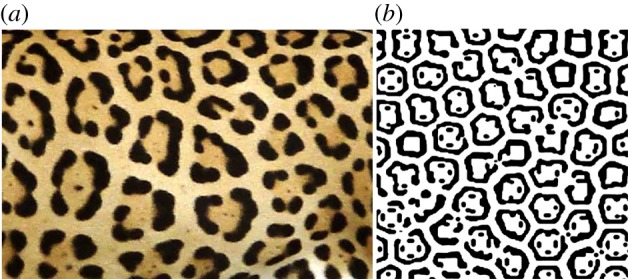
(*a*) The ‘rosette’ spots of a jaguar, and (*b*) an analogous pattern produced by two coupled activator–inhibitor processes. (*b*) Courtesy of Philip Maini, University of Oxford. From [19], © American Physical Society.

More recently, Turing models have been shown capable of reproducing some of the specific fine details of animal markings. For example, two coupled activator–inhibitor processes can produce the broken ring markings characteristic of jaguars (figure 5) [19], and a Turing scheme implemented on the curved shells of ladybirds can produce patterns looking very such like those seen in nature [20].

The stripes of the angelfish offer a particularly suggestive example. These are unusual in that they continue to grow and develop as the fish grow, rather than just being laid down during embryogenesis and then getting blown up like markings on a balloon. The detailed changes in these patterns, such as a characteristic ‘unzipping’ of two merging stripes, is perfectly mimicked by a Turing model [21]. However, later work with the similar patterning system of the zebrafish has shown that although this pattern does seem to result from autoactivation and long-range inhibition between the two pigmented cell types (black melanophores and yellow xanthophores)—making it a genuine Turing pattern—these interactions do not depend on the diffusion of morphogens, but result from the properties of the network of direct cell–cell interaction [22].

**Figure 6.**
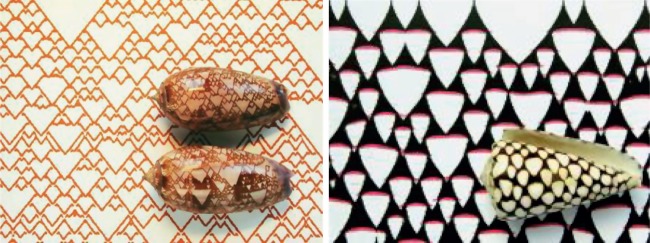
Patterns on seashells and their analogues in theoretical activator–inhibitor systems. From [23], courtesy of Hans Meinhardt, MPI for Developmental Biology, Tübingen.

Turing's *travelling* waves might also produce pigmentation patterns. Meinhardt has shown that an activator–inhibitor scheme with a third morphogen that creates short-ranged but long-lasting inhibition can reproduce the kinds of complex patterns seen on some mollusc shells, which are in effect frozen traces of two-dimensional travelling waves on the rim of the growing shell (figure 6) [23].

It is worth pointing out that there is still no real consensus on what many of these animal markings are for. The default assumption has tended to be that they conceal the animal as camouflage, but this is by no means obviously true in many cases. Some molluscs, for example, spend much of their life cycles covered in mud, with the pigmentation invisible. Meinhardt speculates that here pigmentation might be a side-effect of mechanisms for removing wastes from the mollusc itself.

Even for the zebra, the classic example of a striking quasi-regular pigmentation pattern, it is not clear that the markings are for concealment. Several other functions of the stripes have been proposed, ranging from heat regulation to deterrence of biting insects [24,25]. For wildcats there does seem to be a correlation between those with spots or other markings and a habitat with a variegated appearance—as Rudyard Kipling put it in the *Just so stories* (1902), ‘full of trees and bushes and stripy, speckly, patchy–blatchy shadows'. But there are also outliers, such as the cheetah, which are spotted despite a preference for open habitats. It also seems that the available ‘pattern space’ provided by a Turing-type model accounts fairly well for the range of markings seen in 32 species of wildcats [26]. But it remains unclear how well the precise details of the skin patterns match the visual appearance of the environment in which the animals dwell—in other words, how ‘carefully’ evolution is selecting from the available palette, if indeed that is what is going on.

## 5. But are they real?

**Figure 7.**
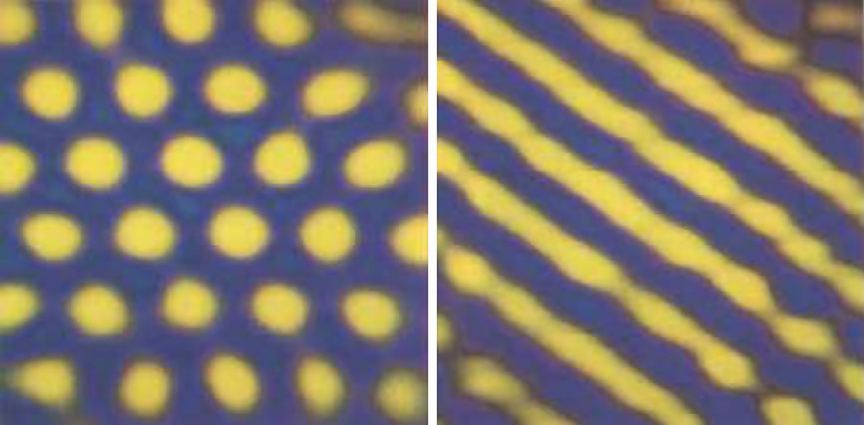
Turing structures in the CIMA reactions: spots and stripes. From [29] courtesy of Harry Swinney, University of Texas at Austin, and Qi Ouyang, Peking University.

Even in purely chemical systems, Turing patterns proved elusive for long after they were proposed. It was not until 1990 that they were first reported in a real chemical system: an oscillating reaction somewhat similar to the BZ reaction, known as the chlorite–iodide–malonic acid (CIMA) reaction. Patrick De Kepper, Jacques Boissonade and their collaborators at the University of Bordeaux saw a band of stationary spots develop in a strip of gel into which the CIMA reagents diffused from opposite sides [27,28]. The following year, extended Turing structures were generated in a two-dimensional layer of the CIMA mixture, and switching between spots and stripes was achieved by altering the concentrations of the reagents (figure 7) [29].

In biology, it turns out that Turing's mechanism is in fact not generally necessary to break the symmetry of a fertilized egg—in many organisms this is disrupted from the outset by maternal proteins diffusing from one side of the embryo. There is evidently a great deal more symmetry-breaking that must happen for a zygote to acquire its full body plan—but it remains far from clear that Turing patterns have much to do with this. For example, the stripe patterns of protein expression that appear in the fruitfly embryo look superficially like Turing's stripes, but they are not generated by the self-organization inherent in his model. Instead there is a hierarchical cascade of patterning steps involving the straightforward diffusion of morphogenetic proteins. Here, a concentration threshold for activating particular genes converts a smooth gradient into an abrupt interface, which becomes a more complex pattern by sequential elaboration of the same simple mechanism [30,31].

For such reasons, until recently Turing's work had rather little impact on developmental biology outside the special case of animal pigmentation. Over the past decade or so, however, good evidence has emerged that Turing patterns and related reaction–diffusion mechanisms do feature in other patterning processes during developmental morphogenesis [32]. For example, the proteins Nodal and Lefty appear to operate as an activator–inhibitor pair during the induction of the mesoderm of metazoans, as demonstrated in experiments on zebrafish and mice [33,34]. And there is some evidence that the hair follicles of mice are positioned in their quasi-regular array on the skin by a process of activation and inhibition involving proteins called Wnt (the activator of follicle formation) and Dkk2 and Dkk4 (inhibitors of Wnt) [35]. For example, genetic mutant mice that produce Dkk proteins in abnormally high amounts develop follicle patterns that match those predicted theoretically from Turing-style activator–inhibitor models of the diffusion and interaction of Wnt and Dkk. However, the details of this patterning process seem likely to be complex, involving various networks of protein and gene interactions rather than a simple activator–inhibitor pair. Something analogous to the patterning of hair follicles may also be at work in the regular arrangement of feathers in birds and the scales of lizards and butterfly wings.

The diverse family of Wnt-type developmental proteins seem likely to produce a range of different patterning mechanisms. Meinhardt [36] has argued that one such protein morphogen organizes the formation of tentacles around the cylindrical gastric column of the hydra—essentially the ring symmetry explored by Turing. The periodic positioning of bird feather barbs can be explained if the protein product of a gene called *Sonic hedgehog* (Shh)—a common patterning gene in many species—behaves as an activator while the bone morphogenic protein 2 is an inhibitor [37]. Through the interaction of these components, the uniform epithelium of the developing feather bud becomes divided into a series of stripe-like ridges that prefigure its break-up into distinct barbs. Meanwhile, the regularly spaced ridges of the mammalian mouth palette seem to be arranged by a Turing-type reaction–diffusion mechanism involving the proteins fibroblast growth factor and Shh as the activator and inhibitor, respectively—albeit with the possible involvement also of other proteins, including those of the Wnt family [38].

Perhaps the most striking challenge to the predominant view that biological development is largely dictated by smooth, long-range biochemical gradients comes from recent evidence that digit formation in the embryo can be regarded as arising from striped Turing patterns [39]. Here too, Wnt proteins play a role. Digit formation is ultimately under the control of a gene called *SOX9*, which triggers differentiation of soft tissue towards the formation of bone and cartilage. *WNT* gene products and bone morphogenetic proteins (products of *BMP* genes) influence the activity of *SOX9* in a manner described by an activator–inhibitor scheme, so that drug-induced suppression of *WNT* or *BMP* leads to predictable changes in the number or spacing of digits.

The important unanswered question for many of these systems seems to be that, while the central elements of activation and inhibition, and reaction and diffusion, do appear to provide a useful basis for framing the problem theoretically, to what extent can the details be reduced and simplified to the two-component scheme proposed by Turing?

## 6. Blossoming theory

Turing's discussion of the whorled leaf arrangements of the woodruff plant in his 1952 paper shows that he suspected his theory might have something to say about a biological patterning process quite different from morphogenesis of the embryo, namely phyllotaxis: the arrangement of leaves or related features on plant stems. He promised that ‘the morphogen theory of phyllotaxis' would be ‘described in a later paper’, and he had already drafted that paper by the time of his death—which, however, prevented its publication at that time [40].^1^ Turing's basic idea was that an activator–inhibitor system of hormones acting at the growing tip of a plant lays down the spots that grow into buds on the cylindrical stem. This seems plausible in the light of current knowledge of plant biology. It has been known since the 1930s that the plant hormone auxin can function as an activator to initiate the growth of new leaf buds (primordia). In 2003 it was shown that phyllotaxis is regulated by proteins that ferry auxin through the outer ‘skin’ of the stem up towards its apex [41]. Existing leaf buds soak up auxin and thus act as sinks, inhibiting the formation of any new buds nearby.

**Figure 8.**
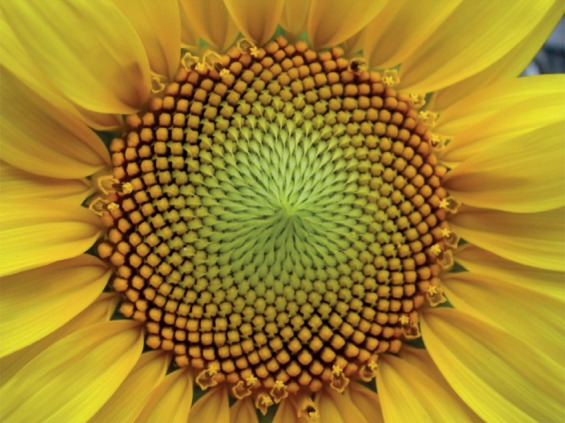
The spiral arrangement of florets or leaf-related features on plants follows the Fibonacci series, as shown here for a sunflower: there are 21 anticlockwise spirals and 34 clockwise spirals. Image: Esdras Calderan/Wikimedia Commons, used under Creative Commons licence.

Phyllotaxis is one of the oldest and most compelling problems of biological pattern formation, not least because it has a mathematical character that seems at first deeply mysterious. The leaves or florets are typically arranged around the stem in a spiral pattern, and when this is projected onto a horizontal plane—as it is in the plant itself for the arrangement of florets in the head of a sunflower or daisy (figure 8)—one finds that there are in fact two groups of counter-rotating spirals. In each of the two groups, the numbers of spirals are always successive numbers in the Fibonacci series, generated from the pair {0,1} by adding together the two preceding numbers: 0, 1, 1, 2, 3, 5, 8, 13, 21, 34 …

Why should the spirals obey this rule? A common view is that the Fibonacci arrangement enables the most efficient packing of the florets. This may well be true, but it does not explain the mechanics of how the growing plant ‘finds' this solution, any more than explaining the leopard's spots in terms of camouflage accounts for how the pigmented spots actually form on the leopard foetus. It seems conceivable, at least, that Turing's scheme might provide the biochemical mechanism: a Turing process involving two sets of activators and inhibitors operating on a cylindrical stem may produce primordia in a (2,3) spiral phyllotaxis pattern [42]. Whether the model can generate higher-order Fibonacci spirals is not clear, however, and there is as yet no direct evidence that such a double activator–inhibitor process really operates in plants.

## 7. Sand, cemeteries and crime

**Figure 9.**
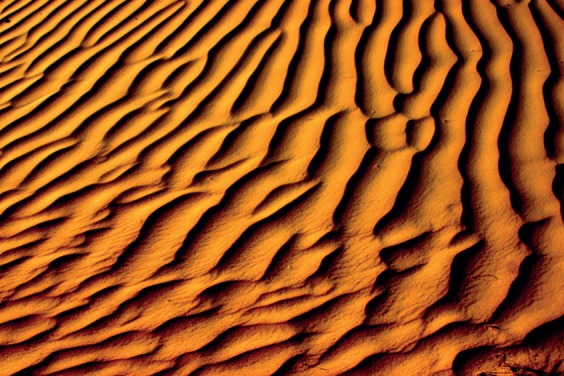
Sand ripples can be regarded as a kind of Turing pattern. Image: EVO, used under Creative Commons licence.

Turing's stripe patterns resemble not only the skin of a zebra, tiger or angelfish, but also some patterns in inanimate nature, such as the ripples in wind-blown sand (figure 9). This may be no coincidence. Meinhardt [23] suggests that, at root, the formation of these sand patterns is akin to an activator–inhibitor system. The mounds and ridges of sand are formed by deposition of wind-blown grains. As a ridge gets bigger, it enhances its own growth by capturing more sand from the moving air. But in doing so it acts as a sink, removing sand from the wind and suppressing the formation of other ripples nearby. The balance between these two processes establishes a roughly constant mean distance between ripples.

The feedbacks involved in replication, competition and predation might set up Turing-type patterns in animal and plant communities. These might account for the patchiness of zooplankton in the sea and the phytoplankton on which they graze [43]. And there is rather compelling evidence that a Turing-type mechanism accounts for structures formed by a species of ant [44]. Mediterranean *Messor sancta* ants collect the bodies of expired colony members and arrange them in piles. The ants constantly pick up and redistribute the corpses, producing a kind of ‘diffusion’ of bodies. Nonetheless, after a certain time the locations of the piles stay fixed. Because ants are more likely to drop a body on a pile as the pile gets larger, there is a positive feedback (activation) controlling their growth, analogous to that by which sand ripples form. There is also long-range inhibition, because the region surrounding a big pile gets swept clear of bodies, making it less likely for a new one to be started in the vicinity. The result is a series of stationary clusters of corpses, and if the ants are confined in a Petri dish then these clusters are created around the perimeter, with precisely the ring geometry that Turing first explored in 1952 (figure 2). Mechanisms like this might underlie many other aspects of habitat formation and grouping, such as nest construction, in higher organisms.

Even human communities, orchestrated by social feedbacks on behaviour and movement, might organize themselves into Turing patterns. A reaction–diffusion model has been proposed to explain the phenomenon of crime hotspots: districts in which the crime rate is anomalously high [45]. Here criminal offenders are modelled as predators who seek ‘prey’ (victims), while both agents move around (diffuse) in the available space. The ‘reaction’—predation of criminals on victims—can be potentially suppressed by an inhibiting agency such as a security measure or a police force.

**Figure 10.**
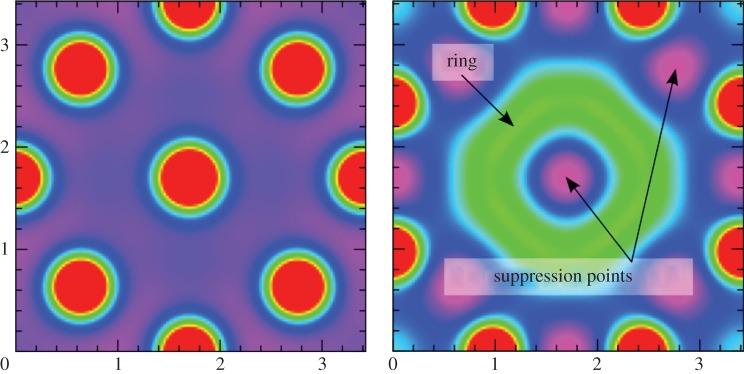
Crime hotspots as Turing structures in a theoretical model of how crime propagates in communities. (*a*) The hotspots in red. If policing is concentrated on one of these spots in an effort to suppress crime, the criminality merely spreads elsewhere in a diffuse ring (green, (*b*)). From [45], courtesy of Martin Short, University of California at Los Angeles.

This model produces two types of hotspots through the competing influences of activation and inhibition. The first are aggregates of individual crimes with overlapping spheres of influence. The second type of hotspot is caused more directly by positive feedback: by the known phenomenon in which crime induces more crime (figure 10). The first sort of hotspot can be eradicated completely by a sufficiently strong inhibiting influence: that is, by locally concentrated policing. But the second kind, corresponding to Turing-type patterns, is harder to eliminate. Focused inhibition may merely cause these hotspots to move or mutate, breaking up into smaller spots or rings in the close vicinity. If this picture is an accurate reflection of the world, it suggests that not all hotspots will yield to the same style of policing, but that different strategies might be needed in different situations.

## 8. Conclusion

Alan Turing's 1952 paper, proposed by an author with no real professional background in the subject he was addressing, put forward an astonishingly rich idea. The formation of regular structures by the competition between an autocatalytic activating process and an inhibiting influence, both of which may diffuse through space, now appears to have possible relevance not just for developmental biology but for pure and applied chemistry, geomorphology, plant biology, ecology, sociology and perhaps even astrophysics (a reaction–diffusion mechanism has, for example, been suggested as the origin of spiral galaxies). Turing seems to have identified one of nature's general mechanisms for generating order from macroscopic uniformity and microscopic disorder. Several of the putative Turing structures in nature remain speculative, and indeed, it is notoriously difficult to distinguish between different candidate processes for generating a particular pattern. But, there seems little question that nature, including the living world, does use Turing's mechanism as one way of producing its rich and often beautiful panoply of forms.

From a biological perspective, the broader question is how a spontaneous process such as that deduced by Turing, which gives rise to a particular palette of shapes and patterns, interacts with natural selection. To what extent can evolution adapt and modify Turing structures? Are all such structures necessarily adaptive at all? Or are we too readily tempted, when we descry order and regularity in nature, to attribute a ‘purpose’ to it? Might some of it, at least, represent nothing more than a kind of intrinsic creative potential in the natural world?

## Endnotes

1 It was published, along with his ‘Outline of the development of a daisy’, in *Morphogenesis: collected works of A. M. Turing*, vol. 3 (ed. P. T. Saunders). Elsevier North-Holland, 1992.

## Author profile

**Philip Ball** is a freelance writer. He worked as an editor at *Nature* for many years, and now writes on all areas of science, and in particular on interactions between science, art and culture. His books include *The self-made tapestry* (Oxford University Press, 1998), a comprehensive survey of natural pattern formation, which was reissued in 2009 as a trilogy called *Nature's patterns*, comprising *Shapes*, *Flow* and *Branches* (Oxford University Press). His most recent book is *Invisible: the dangerous allure of the unseen* (2014).
